# Differential effects of plant-beneficial fungi on the attraction of the egg parasitoid *Trissolcus basalis* in response to *Nezara viridula* egg deposition

**DOI:** 10.1371/journal.pone.0304220

**Published:** 2024-05-21

**Authors:** Sara Van Hee, Tuğcan Alınç, Berhane T. Weldegergis, Marcel Dicke, Stefano Colazza, Ezio Peri, Hans Jacquemyn, Antonino Cusumano, Bart Lievens

**Affiliations:** 1 CMPG Laboratory for Process Microbial Ecology and Bioinspirational Management (PME&BIM), Department of Microbial and Molecular Systems (M2S), KU Leuven, Leuven, Belgium; 2 Leuven Plant Institute (LPI), KU Leuven, Leuven, Belgium; 3 Department of Agricultural, Food and Forest Sciences, University of Palermo, Palermo, Italy; 4 Laboratory of Entomology, Wageningen University, Wageningen, The Netherlands; 5 Laboratory of Plant Conservation and Population Biology, Biology Department, KU Leuven, Leuven, Belgium; University of Carthage, TUNISIA

## Abstract

There is increasing evidence that plant-associated microorganisms play important roles in defending plants against insect herbivores through both direct and indirect mechanisms. While previous research has shown that these microbes can modify the behaviour and performance of insect herbivores and their natural enemies, little is known about their effect on egg parasitoids which utilize oviposition-induced plant volatiles to locate their hosts. In this study, we investigated how root inoculation of sweet pepper (*Capsicum annuum*) with the plant-beneficial fungi *Beauveria bassiana* ARSEF 3097 or *Trichoderma harzianum* T22 influences the olfactory behaviour of the egg parasitoid *Trissolcus basalis* following egg deposition by its host *Nezara viridula*. Olfactometer assays showed that inoculation by *T*. *harzianum* significantly enhanced the attraction of the egg parasitoid, while *B*. *bassiana* had the opposite effect. However, no variation was observed in the chemical composition of plant volatiles. Additionally, fitness-related traits of the parasitoids (wasp body size) were not altered by any of the two fungi, suggesting that fungal inoculation did not indirectly affect host quality. Altogether, our results indicate that plant inoculation with *T*. *harzianum* T22 can be used to enhance attraction of egg parasitoids, which could be a promising strategy in manipulating early plant responses against pest species and improving sustainable crop protection. From a more fundamental point of view, our findings highlight the importance of taking into account the role of microorganisms when studying the intricate interactions between plants, herbivores and their associated egg parasitoids.

## Introduction

Plants engage in multiple biotic interactions that profoundly influence the relationships between plants and insects [1–3]. Most plants live in close association with diverse microorganisms, including bacteria and fungi, several of which play a crucial role in the interactions between plants and insect herbivores [4, 5]. Plant-beneficial microorganisms like mycorrhizal fungi, plant-growth promoting rhizobacteria or plant-growth promoting fungi not only enhance plant growth [6–8], but also protect plants against herbivores through direct and indirect mechanisms [9–13]. Direct effects may result from the production of deterrents, antifeedants or toxins, exerting direct negative effects on the behaviour or performance of the herbivorous insects [14, 15]. Indirect effects may result from enhanced recruitment of natural enemies of herbivores or an improvement of their activity [16, 17].

To locate vital resources such as food, mates or oviposition sites, insects are equipped with a variety of sensory systems, allowing them to perceive and interpret information from their environment encoded as olfactory, gustatory, acoustic, tactile and visual cues [18]. In their search for food plants, herbivores predominantly rely on direct cues associated with their host plant, such as odours and colours [18]. In contrast, the natural enemies of these herbivores, including predators and parasitoids, rely heavily on the indirect information from plant volatile organic compounds (VOCs) induced by herbivore feeding or egg deposition to locate their (often concealed) prey or hosts, commonly referred to as “herbivore-induced plant volatiles” (HIPVs) and “oviposition-induced plant volatiles” (OIPVs) respectively [19–22].

There is increasing evidence that plant-associated microbes can alter the production of plant volatiles and, hence, modify plant-insect interactions [1, 4, 23]. For example, some studies have documented enhanced herbivore-repellent characteristics in microbe-inoculated plants, resulting in diminished feeding damage [11, 24], while others have reported increased herbivore attraction [25, 26]. Plant-associated microbes have also been shown to prime or alter plant defence pathways involved in indirect plant defences [17], thereby increasing the attraction of natural enemies towards herbivore-infested plants [9, 12, 27–29]. However, their effects do not appear to be exclusively positive [17]. For instance, reduced host location by parasitoids, reduced parasitism efficiency or reduced parasitoid emergence have been reported as well on plants treated with root-colonizing microorganisms [30–33]. Although the mechanisms behind these observations are not yet completely clear, it is evident that microbial colonization may affect the plant chemistry, by influencing the plant’s nutrient uptake [11] or inducing a cascade of changes in plant metabolism [34, 35]. This in turn can also affect parasitoid host quality, and, since parasitoids rely on the quality of their host [36], also parasitoid fitness-related traits [30, 37].

Egg parasitoids are an important group of natural enemies of herbivorous insects as they kill their hosts in the egg stage before plant damage occurs [38, 39]. When searching for hosts, egg parasitoids use host-associated cues, especially OIPVs and odours from gravid females [21, 40, 41]. To date, virtually nothing is known about whether the host-seeking behaviour of egg parasitoids can be modulated by plant-associated microbes. From a biocontrol perspective, this is remarkable as enhancing the attraction of egg parasitoids towards egg-infested plants may increase their biocontrol efficacy, especially since host eggs are only available for a short period of time [42].

The main aim of this study was to investigate whether root inoculation with plant-beneficial fungi enhances the attraction of egg parasitoids after egg deposition by altering the plant VOC composition, and how this is mediated by fungal species. Specifically, we evaluated the effects of root-inoculation of sweet pepper (*Capsicum annuum* L.; Solanaceae) with the fungal strains *Beauveria bassiana* ARSEF 3097 (Hypocreales: Cordycipitaceae) and *Trichoderma harzianum* T22 (Hypocreales: Hypocreaceae) on the olfactory responses of the egg parasitoid *Trissolcus basalis* (Wollaston) (Hymenoptera: Scelionidae) to plants infested with eggs of the southern green stink bug *Nezara viridula* (Linnaeus) (Hemiptera: Pentatomidae). *Trichoderma* spp. are well known for their capacity to stimulate plant growth and protect plants against pests and diseases [43, 44]. By contrast, the indirect plant protective capabilities of entomopathogenic fungi that establish endophytic associations with plants like *B*. *bassiana* have only recently been discovered [15, 45, 46], but their full potential still remains to be revealed. *Nezara viridula* is responsible for severe damage to major open field crops such as soybean and cotton, and has become an important pest in greenhouses in Northwestern Europe, where it causes significant damage to tomato, sweet pepper, eggplant and cucumber [47]. *Trissolcus basalis* is the main egg parasitoid of *N*. *viridula*, and has emerged as a promising candidate to control the pest in both open fields and greenhouses [47, 48]. Nevertheless, given the observed variability in its effectiveness as a biocontrol agent [48–50], enhancing its attraction to egg-infested plants by applying beneficial microorganisms could serve as an effective strategy to achieve more consistent and reliable outcomes. First, we asked whether fungal inoculation affected parasitoid olfactory behaviour following stink bug oviposition. As a comparison, stink bug feeding without oviposition was included. Subsequently, we studied the plant’s headspace composition to see if fungal inoculation affected the chemical composition of induced plant volatiles. Finally, as microbial colonization may have cascading effects and impact parasitoid fitness-related traits, we also investigated whether fungal inoculation affected parasitoid fitness-related features through body size measurements.

## Materials & methods

### Study organisms

*Trichoderma harzianum* T22 (recently re-classified as *Trichoderma afroharzianum* [51]; for consistency with previous studies further referred to as *T*. *harzianum*) is the active ingredient in several biopesticides and biofertilizers such as Trianum-P (Koppert Biological Systems, The Netherlands) from which it was isolated [52, 53]. *Beauveria bassiana* ARSEF 3097 was acquired from the Agricultural Research Service Collection of Entomopathogenic Fungal Cultures (ARSEF; New York, USA), and represents the active ingredient in several commercially available bioinsecticides such as Naturalis^®^ (Intrachem, Italy). The strain can colonize diverse plant species endophytically following artificial inoculation, including sweet pepper, besides its direct entomopathogenic capability [25, 54]. The fungal strains were preserved at -80°C in 35% glycerol on potato dextrose agar (PDA) plugs, until used in the experiments. For all experiments, sweet pepper (*Capsicum annuum* L.) cv ‘IDS RZ F1’ (Rijk Zwaan, The Netherlands) was used. Plants were grown in a 3:1 mixture of potting mix (Universal potting mix; Agrofino, Belgium) and perlite under controlled conditions in a climate cabinet (MD1400, Snijders Labs, The Netherlands) at 23 ± 1˚C, 65 ± 2% RH and a 16L:8D photoperiod, with white LED lights to provide a photosynthetic flux density of 790 μmol photons m^-2^ s^-1^.

*Nezara viridula* was reared in insect cages (47.5 × 47.5 × 47.5 cm) (BugDorm, MegaView Science Co. Ltd., Taiwan) under controlled conditions (ECL02, Snijders Labs, The Netherlands) at 25 ± 1°C, 70 ± 2% RH and a 16L:8D photoperiod. The population was established from individuals obtained from the University of Palermo [10], to which yearly newly field-caught specimens from Flanders (Belgium) were introduced. Stink bugs were fed with seasonal organic vegetables (such as tomatoes, cabbage and beans) and organic seeds (sunflower, soybean and peanut). A wet cotton roll served as a water source, while sweet pepper plants along with paper towels were provided as oviposition substrate. Food and water were replaced every two to three days, and newly laid eggs were systematically collected by carefully removing the egg masses from the paper towels, cage mesh or sweet pepper leaves. Both the eggs and *N*. *viridula* nymphs that emerged from the collected eggs were kept under the same conditions as described above, while adults were utilized for the continuation of the rearing and the experiments.

*Trissolcus basalis* individuals were kept in 50 mL polypropylene vials with a cotton plug (VWR International, USA) under the same conditions as described for *N*. *viridula*. Every two days a few drops of honey water (80:20 v/v) were provided on the cotton plug as a food source. To maintain the parasitoid colony, *N*. *viridula* egg masses were individually exposed to two mated *T*. *basalis* females. Parasitized egg masses were then placed in new vials until the emergence of the wasps. Twenty-four hours prior to the experiments, female *T*. *basalis* that were 5–7 days old were individually transferred into small 6 mL glass vials sealed with a cotton plug, and incubated as described above (supplemented with a drop of honey water on a piece of parafilm). Subsequently, parasitoids were transferred to the bioassay room and allowed to acclimate for approximately 1 h before the tests. To avoid bias from parasitoids reared on different egg masses, individuals from different egg masses were randomized over the different treatments.

### Fungal inoculation and treatments

The preparation of fungal spore suspensions and subsequent plant inoculation were performed as outlined previously [6, 25]. In short, stored agar plugs from the fungi were plated on PDA (Oxoid Holdings Ltd., United Kingdom) (*T*. *harzianum* T22) or Sabouraud dextrose agar medium supplemented with 0.25% yeast extract (SDAY) (Oxoid Holdings Ltd., United Kingdom) (*B*. *bassiana* ARSEF 3097), and incubated at 25°C for seven days. Next, sterile physiological saline solution (0.8% NaCl) was added to the plates, and the spores were carefully scraped off to prepare the fungal spore suspensions. To eliminate mycelial fragments, the suspensions were filtered through a microcloth (Mira Cloth, Merck, USA) and washed three times with physiological saline solution. After determining the concentration of conidia using a Bürker hemocytometer, the spore suspensions were diluted to a final concentration of 1 × 10^7^ conidia mL^-1^. Before conducting the experiments, conidial viability was checked by plating an aliquot of 100 μL of 1 × 10^3^ conidia mL^-1^ on three agar plates and counting the numbers of germinated and ungerminated conidia under the microscope after incubation at 25°C for 24 h. Spores were considered germinated when the germ tube was at least two times longer than the spore diameter. The germination tests demonstrated > 90% viability rate for all conidial suspensions used. Plant inoculation was performed when the plants had reached the first true leaf stage (BBCH stage 101). Therefore, after rinsing the seedling roots with tap water, the roots were submerged for 18 h in 10 mL of either the conidial spore suspension (Bb, *B*. *bassiana*; Th, *T*. *harzianum*) or physiological saline solution to obtain non-inoculated, control plants (Co). Subsequently, the seedlings were transplanted in 11 cm diameter plastic pots filled with the same potting mixture as mentioned above. Plants were then put in controlled conditions as mentioned before, ensuring that plants subjected to different treatments could not make any contact with each other.

To perform the experiments, four weeks after inoculation, fungus-inoculated and non-inoculated plants (ca. six weeks old) were individually exposed to two gravid *N*. *viridula* females or were left untouched. Female stink bugs were allowed to feed only (F) or feed and oviposit, hereafter simply referred to as oviposit (O). Stinkbugs are known to feed on plant tissues while laying eggs [55]. Hence, this resulted in three treatments: (1) feeding, (2) oviposition, and (3) no infestation. For the first two treatments, plants were exposed to stink bugs in cages (30 × 30 × 30 cm) under controlled conditions (23 ± 1˚C, 65 ± 2% RH and a 16L:8D photoperiod), while the uninfested plants were incubated in cages under the same conditions. In both the feeding and oviposition treatments stink bug feeding was confirmed by visual observation, with females consistently observed inserting their stylets into leaf tissue. Plants were used in the experiments 24 h after exposure to feeding or within 24 h after discovery of an egg mass (inspected daily). On average, egg masses contained approximately 80 eggs.

### Two-choice olfactometer bioassays

The olfactory responses of *T*. *basalis* were assessed in a two-choice Y-tube olfactometer [56] made from a polycarbonate body (stem 9 cm; arms 8 cm at a 130° angle; internal diameter 1.5 cm) sandwiched between two glass plates. Charcoal-filtered air at a flow rate of 0.8 L min^-1^ was pumped into each of the olfactometer arms by passing a glass bottle (diameter 12 cm; height 52 cm) containing a test plant as odour source. Plants were carefully placed in the glass bottles after wrapping the pot and soil with aluminium foil to limit belowground VOCs from moving into the headspace and impact parasitoid behaviour. The Y-tube olfactometer was positioned at an incline of 20° and homogeneously illuminated from above by four 24 W T5 TL-fluorescent tubes (16 × 549 mm, 1350 lumen, 5500 K) to stimulate movement of the insects towards the bifurcation. The table was closed off by white curtains, to exclude any visual bias from the surroundings [56]. A single female wasp was introduced at the entrance of the Y-tube and allowed to walk freely in the olfactometer. Next, the olfactory behaviour of the introduced wasps was recorded for ten minutes using a camera (Logitech C920), and the recorded videos were analysed by CowLog 3 software [57]. Wasp responses were recorded for 10 min and assessed in terms of residence time, i.e., the time spent by the wasps in each arm after crossing a virtual line, defined at 5 mm distal to the bifurcation of the Y-tube olfactometer. In the Y-tube olfactometer, females of *T*. *basalis* have the tendency to explore first the entire area and then spend more time in the arm with the most attractive odour. Thus, olfactory responses for this species are better predicted by analysing residence time data rather than first-choice (first arm chosen) data as often done for other parasitoids [55, 58, 59]. Each bioassay was replicated using five pairs of plants, and ten female wasps per pairwise combination were tested (in total 50 wasps). Each wasp was only tested once. The position of the plants was switched after testing five wasps in order to identify any unforeseen bias in the setup. In total, six pairwise combinations were tested, as presented in Table 1. At the end of the assay, all polycarbonate olfactometer parts were rinsed with fragrance-free laboratory detergent and deionized water, followed by air-drying. The glass components were rinsed with deionized water and acetone, after which they were incubated at 175°C overnight. All bioassays were conducted at 25 ± 2°C, 65 ± 5% RH, and were performed between 09:00 and 18:00. Experiments were set up randomized over several days.

**Table 1:** Overview of the pairwise combinations tested in Y-tube olfactometer assays.

Comparison	Odour source 1	Odour source 2
(1): Co *versus* Co_O	Non-inoculated control plant	Non-inoculated plant subjected to stink bug oviposition
(2): Co *versus* Co_F	Non-inoculated control plant	Non-inoculated plant subjected to stink bug feeding
(3): Co_F *versus* Bb_F	Non-inoculated plant subjected to stink bug feeding	*Beauveria bassiana*-inoculated plant subjected to stink bug feeding
(4): Co_F *versus* Th_F	Non-inoculated plant subjected to stink bug feeding	*Trichoderma harzianum*-inoculated plant subjected to stink bug feeding
(5): Co_O *versus* Bb_O	Non-inoculated plant subjected to stink bug oviposition	*Beauveria bassiana*-inoculated plant subjected to stink bug oviposition
(6): Co_O *versus* Th_O	Non-inoculated plant subjected to stink bug oviposition	*Trichoderma harzianum*-inoculated plant subjected to stink bug oviposition

### Dynamic headspace sampling and analysis of volatile organic compounds

For each treatment, volatiles were collected by dynamic headspace sampling to assess the VOC composition of the aboveground plant parts according to the procedures described in Wilberts *et al*. [25] with a few modifications. Volatiles were sampled from a different set of plants (*n* = 9–10) of the same age and treated in a manner identical to those that were tested in the Y-tube olfactometer bioassays. Plants were placed individually in a glass dome with a height of 20 cm and a diameter of 23 cm. The dome was sealed using aluminium plates around the stem without constricting the plants. To avoid external VOCs from entering the system, charcoal-filtered air was pumped in at a rate of 300 mL min^-1^, while simultaneously air was drawn out at a rate of 200 mL min^-1^ through a stainless steel tube (89 mm length; 6.4 mm outer diameter; 5 mm inner diameter) filled with 200 mg Tenax TA adsorbent (20/35 mesh; 165 CAMSCO, Houston, TX, USA), so that positive pressure was maintained within the dome. Collections were carried out under controlled laboratory conditions (23 ± 2°C; 65 ± 5% RH) for a period of 3 h (lights on), after acclimatization of the plants to the room and the glass domes for 30 min. Between subsequent volatile collections, the glass domes were rinsed with water and acetone and incubated at 175°C for 2 h. After VOC collection, the above-ground parts of the plants were cut and weighed for normalization of the VOC data (see below). Background VOCs from empty glass domes were collected at regular time intervals.

Desorption of volatiles from the Tenax TA, as well as separation and detection of volatiles, was carried out using a Thermal Desorber TD100-xr (Markes International Ltd., UK) connected to a 7890B gas chromatograph (GC) coupled to quadrupole-time-of-flight mass spectrometer (Q-ToF) (both from Agilent Technologies, USA). Volatiles released from the adsorbent at 250°C for 10 min under a helium flow of 30 mL min^-1^ were simultaneously re-collected in an electronically-cooled sorbent trap (Markes International Ltd., UK) at 0°C. Once the desorption and re-collection process was completed, volatile compounds were released from the cold trap by ballistic heating at 40°C s^-1^ to 280°C, which was then kept for 5 min, while volatiles were transferred to a 30 m length × 0.25 mm inner diameter × 1 μm film thickness DB-5MS analytical column (Phenomenex, USA), placed inside the oven of a GC (Agilent Technologies, USA) for further separation. The GC oven temperature was initially held at 40°C for 2 min and was raised at 10°C min^-1^ to 100°C, where it was held for 1 min. The temperature was then raised at 5°C min^-1^ to 140°C and was then immediately raised at 10°C min^-1^ to a final temperature of 280°C, where it was kept for 1 min under a constant helium flow of 1.2 mL min^-1^. Column effluents were ionized by electron impact ionization at 70 eV and detected with an accurate mass Q-ToF MS (Agilent Technologies, USA), acquiring mass spectra from 35–400 m/z at an acquisition rate of 5 spectra s^-1^. The transfer line and ion source of the Q-ToF MS were set at 280 and 230°C, respectively.

Chromatograms recorded for the presence of plant volatile compounds using MassHunter deconvolution software (Agilent Technologies, USA) were converted to Xcalibur data through a two-step raw data conversion program available in MetAlign software [60]. Automated baseline correction, peak selection (S/N > 3), and alignments of all extracted mass signals of the raw data were processed following an untargeted metabolomic workflow using MetAlign software, producing detailed information on the abundance (peak height) of the mass signals representing the available metabolites [60]. Subsequently, the extracted mass features were reconstructed into potential compounds using the MSClust software through data reduction employing unsupervised clustering and extraction of putative metabolite mass spectra [61]. Chromatograms were visually inspected to ensure that true peaks were selected and that no peaks were missing. Tentative identification of volatile metabolites was based on a comparison of the reconstructed mass spectra with those in the NIST 2014 and Wageningen Mass Spectral Database of Natural Products MS libraries, as well as experimentally obtained linear retention indices (LRIs).

### Fitness-related traits of *Trissolcus basalis*

In order to assess effects of fungal inoculation on fitness-related traits of *T*. *basalis*, we measured the proportion of parasitized eggs within a single egg mass, laid on fungus treated and non-inoculated plants. Additionally, the size (i.e. right hind tibia length) of female wasps that successfully emerged from these egg masses was measured. In insects, and especially in hymenopteran parasitoids, adult body size is known to be positively associated with fitness and is commonly used to assess parasitoid performance [62]. Within 24 h after *N*. *viridula* oviposition, one *T*. *basalis* female was introduced in a fine mesh bag enclosing the leaf containing the egg mass. After 24 h, the female parasitoid was removed, and the parasitized egg masses were left on the leaf until right before parasitoid emergence. Then, they were carefully removed from the leaf and were placed into 50 mL plastic vials until the wasps’ emergence. The proportion of the egg mass that was parasitized was recorded, and freshly emerged wasps were killed in 96% ethanol and kept in phosphate-buffered saline (PBS) until further use. Measurements were conducted using a stereoscope (Zeiss SteREO Discovery.V12) with AxioVision SE64 Rel. 4.9.1 software for image acquisition and analysis. A total of ten randomly selected female wasps from five egg masses were included in the experiment for each of the three treatments.

### Statistical analysis

Behavioural data were analysed by linear mixed models (LMMs) with fungal treatment as a fixed factor and each plant pair (with the parasitoid individual nested within the plant pair) as a random factor, to account for pseudoreplication [56]. For each pairwise combination, a separate LMM was used. Significance of the fixed term in the model was determined using likelihood ratio tests (LRTs), whereas model fit was assessed with residual plots [63]. A similar LMM test followed by Tukey’s multiple comparison test was also used to analyse the effect of fungal inoculation on the size of wasps. Prior to statistical analysis of the volatile emissions data, the peak height of each compound was normalized by dividing by the biomass (aboveground fresh weight (g)) of the plant used during VOC collection to account for plant-to-plant variation within treatment groups. To assess whether overall VOC emissions differed between treatments, a permutational multivariate analysis of variance (perMANOVA) was performed on the log-transformed values, based on 1000 permutations, using the adonis2 function of the vegan package [64]. Further, a heatmap of calculated Z-scores was constructed using the heatmap.2 function from the gplots package [65] to visualize differences in the VOC profiles among treatments [65]. Z-scores were calculated for the average of the log-transformed data per treatment, by subtracting the average value of all treatments for the respective compound from the average value of that treatment and dividing by the respective standard deviation. Subsequently, the data were analysed in the same pairwise comparisons as those made in the behavioural assays to explain potential differences in wasp behaviour. Therefore, VOC emissions were visualized by non-metric multidimensional scaling (NMDS) using the Bray-Curtis dissimilarity measure, in the vegan package in R [64]. Additionally, a perMANOVA was performed as described above to assess differences in overall VOC profiles among treatments. Furthermore, for each tested pair of treatments, differences in the peak heights of individual volatile compounds were analysed, after checking assumptions of normality, using a Student’s *t*-test or Wilcoxon rank-sum test (for compounds with a normal or non-normal distribution, respectively) on log-transformed values. To correct for multiple testing, the raw *P* values were adjusted using the Benjamini-Hochberg correction, to control the false discovery rate at 5% [66]. A significance level of α = 0.05 was used to determine significant differences, and results were visualized using the ggplot2 package. All analyses and visualization of the data were performed in R version 3.6.3 [67].

## Results

### Two-choice olfactometer bioassays

**Fig 1:**
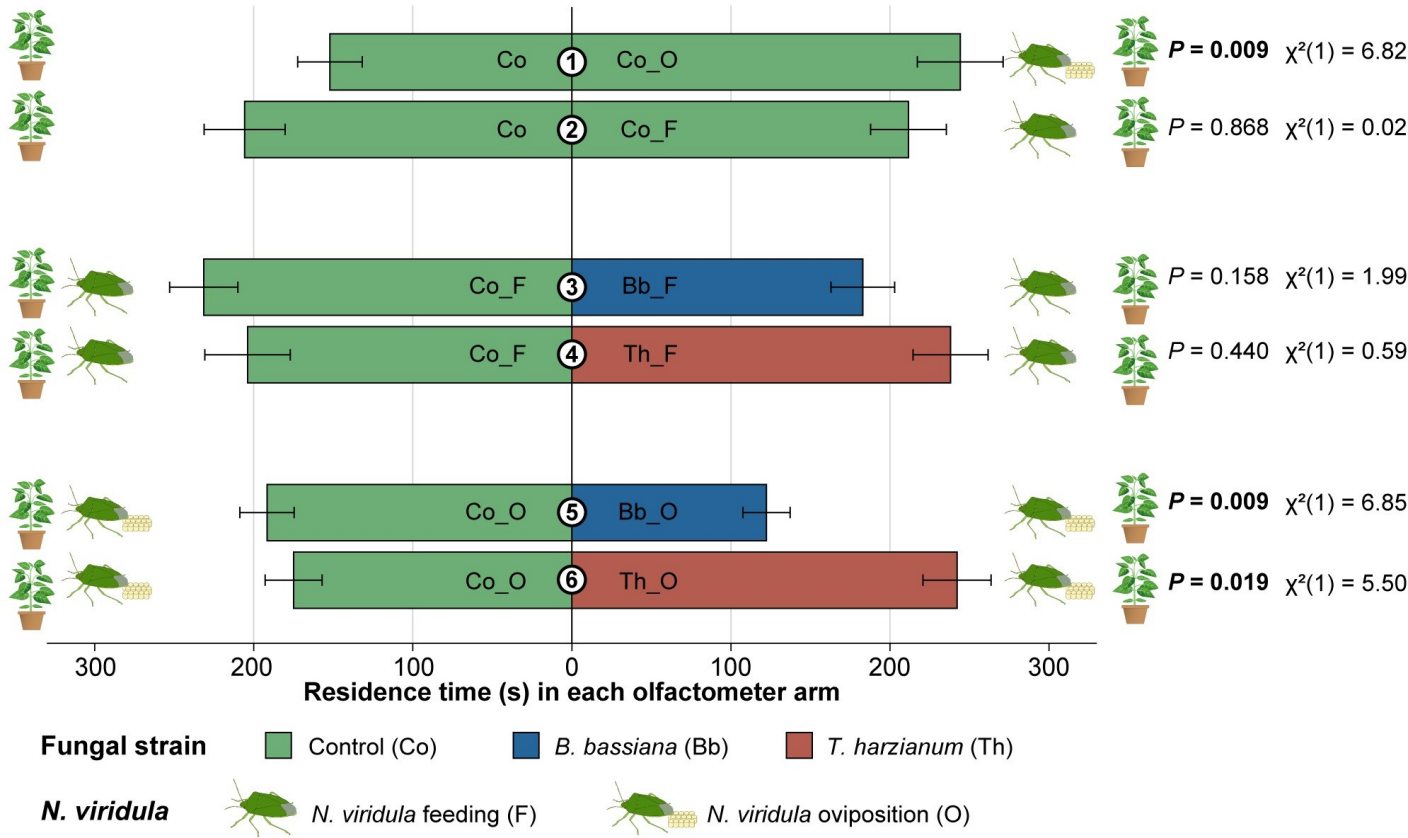
Olfactory behaviour of *Trissolcus basalis* females in a Y-tube olfactometer when given the choice between odour sources coming from differently treated sweet pepper plants. Plants were uninfested or subjected to *Nezara viridula* feeding or oviposition, and inoculated with *Beauveria bassiana* ARSEF 3097 (blue) or *Trichoderma harzianum* T22 (red) or mock-inoculated with physiological saline solution (green). Bars represent the mean (mean ± SE) time spent by the female wasps (*n* = 50) in each olfactometer arm over an observation period of 600 s. *P* values in bold indicate significant differences in the residence times (*P* ≤ 0.05), when compared to a 50:50 distribution (linear mixed model).

In two-choice Y-tube olfactometer bioassays, females of *T*. *basalis* significantly preferred sweet pepper volatiles induced by *N*. *viridula* oviposition over volatiles from non-infested control plants (Co *versus* Co_O: *P* = 0.009) (Fig 1). More specifically, the wasps spent 60.5% more time in the olfactometer arm containing the oviposition-induced plant volatiles. Yet wasps did not show any significant response when given a choice between non-infested control plants and plants subjected to *N*. *viridula* feeding only (Co *versus* Co_F: *P* = 0.868). Likewise, wasps did not discriminate between volatiles from non-inoculated and fungus-inoculated plants that were subjected to feeding, regardless of the identity of the fungal strain (Co_F *versus* Bb_F: *P* = 0.158; Co_F *versus* Th_F: *P* = 0.440). By contrast, *T*. *harzianum* significantly increased the wasps’ preference to volatiles induced by egg deposition compared to the volatiles emitted by non-inoculated plants that were subjected to egg deposition, with wasps spending 38.4% more time in the olfactometer arm from the fungus-inoculated plant (Co_O *versus* Th_O: *P* = 0.019). When eggs were deposited on plants that were inoculated with *B*. *bassiana*, wasps preferred the odour from non-inoculated plants (Co_O *versus* Bb_O: *P* = 0.009). In this case, their residence time in the olfactometer arm containing plant volatiles from *B*. *bassiana*-inoculated plants was 36.2% higher compared to the control arm (Fig 1).

### Volatile composition

**Fig 2:**
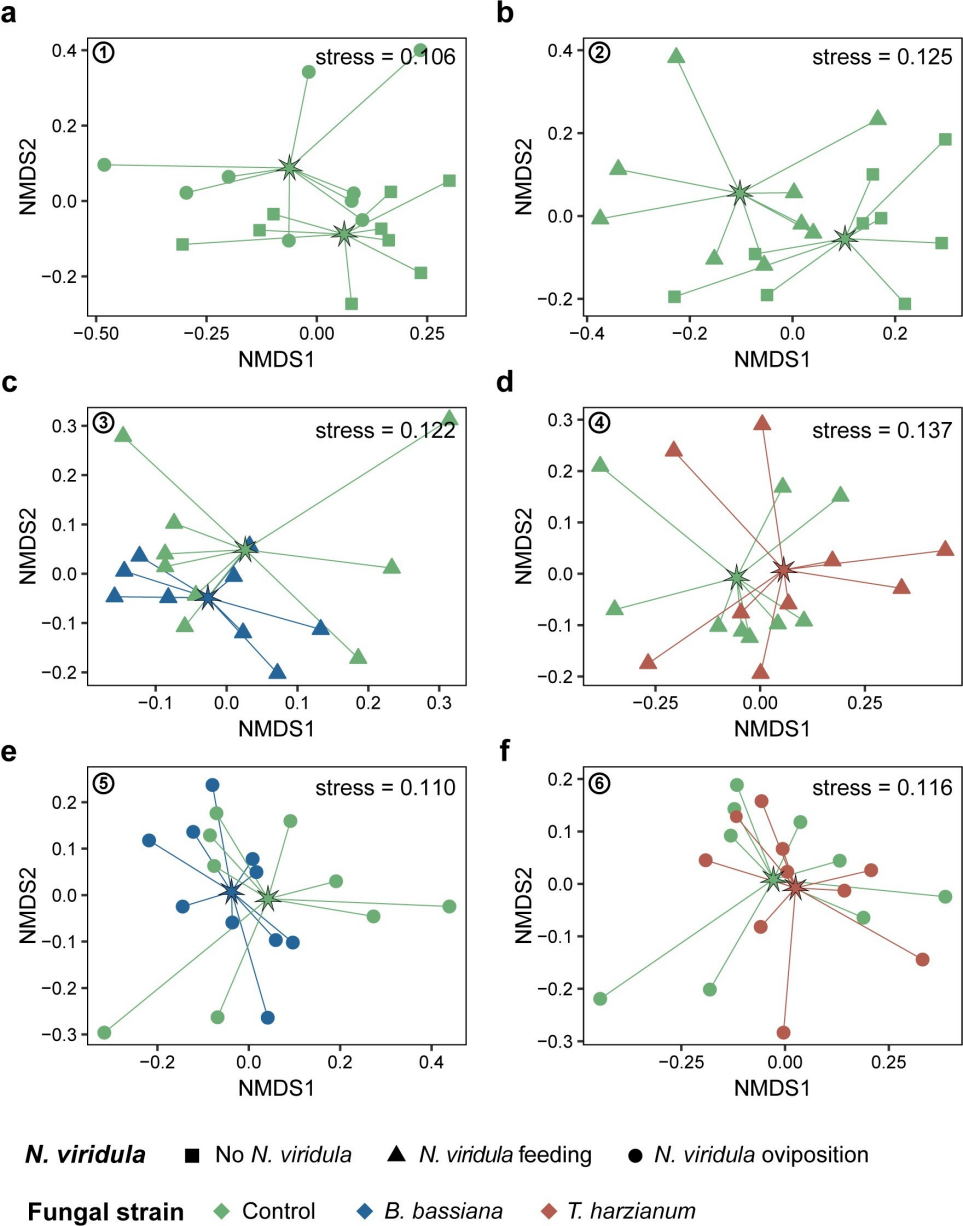
Non-metric multidimensional scaling (NMDS) ordination plots based on Bray-Curtis dissimilarities of plant VOCs emitted by differently treated sweet pepper plants. Plants inoculated with *Beauveria bassiana* ARSEF 3097 (blue) or *Trichoderma harzianum* T22 (red), or mock-inoculated with physiological saline solution (green), were uninfested (square) or subjected to *Nezara viridula* feeding (triangle) or oviposition (circle). Each dot represents an individual sample (*n* = 9–10 per treatment). The star represents the group centroid. The circled numbers correspond to the pairwise comparisons in the Y-tube olfactometer assays (Table 1): **(a)** Co *versus* Co_O, **(b)** Co *versus* Co_F, **(c)** Co_F *versus* Bb_F, **(d)** Co_F *versus* Th_F, **(e)** Co_O *versus* Bb_O, and **(f)** Co_O *versus* Th_O.

Volatile analysis revealed a total of 75 compounds in the headspace of sweet pepper plants subjected to the different treatments (S1 Table and S1 Fig). These compounds belonged to the class of terpenoids (52), nitrogen-containing compounds (3), fatty acid derivatives (7), and benzenoids or phenylpropanoids (13) (S1 Table). PerMANOVA did not indicate any statistical differences in VOC composition between the different treatments when considering all data together (*F*_6,59_ = 1.462, *P* = 0.055). When focusing on the pairs of treatments examined in the olfactometer bioassays, the NMDS ordination plots revealed no clear separation in the VOC composition among the compared treatments (Fig 2). Likewise, perMANOVA did not reveal any significant differences in the VOC profiles among the treatments compared (Co *versus* Co_O: *F*_1,16_ = 1.101, *P* = 0.307, Fig 2A; Co *versus* Co_F: *F*_1,16_ = 1.521, *P* = 0.159, Fig 2B; Co_F *versus* Bb_F: *F*_1,16_ = 1.264, *P* = 0.264, Fig 2C; Co_F *versus* Th_F: *F*_1,16_ = 0.936, *P* = 0.414, Fig 2D; Co_O *versus* Bb_O: *F*_1,16_ = 0.650, *P* = 0.691, Fig 2E; Co_O *versus* Th_O: *F*_1,16_ = 0.567, *P* = 0.750, Fig 2F). When looking at the individual detected VOCs, no significant differences were found after correcting for multiple hypothesis testing (S2 Table).

### Fitness-related traits of *Trissolcus basalis*

**Fig 3:**
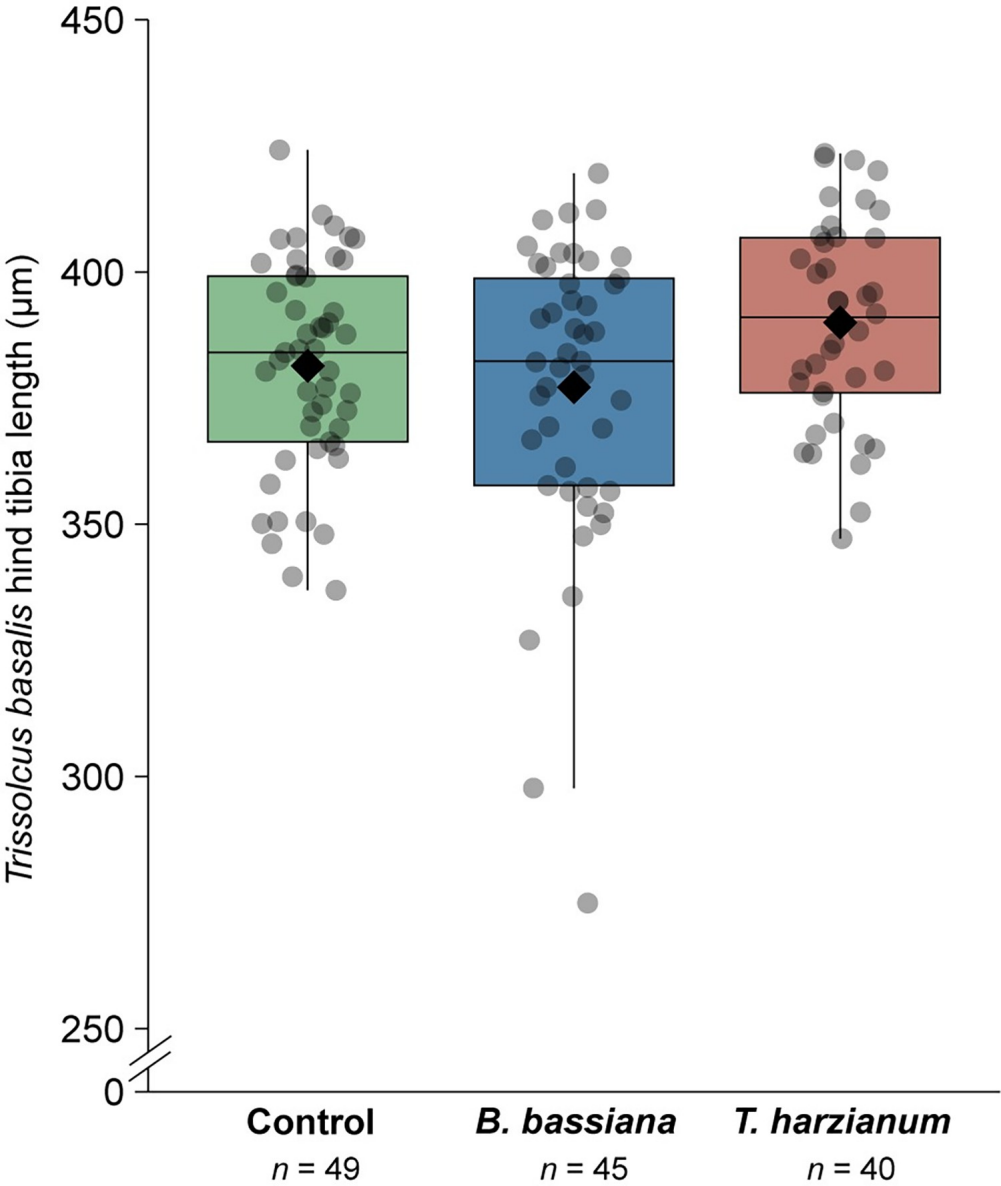
Performance of *Trissolcus basalis* parasitoid individuals (determined by hind tibia length (μm)), emerged from *Nezara viridula* egg masses deposited on plants inoculated with *Beauveria bassiana* ARSEF 3097 (blue) or *Trichoderma harzianum* T22 (red), or mock-inoculated with physiological saline solution (green). The lower, middle and upper lines of the boxplots correspond to the first quartile, median and third quartile, respectively, while the diamond represents the average.

Fungal inoculation did not affect the size of *T*. *basalis* parasitoid females that emerged from the egg masses deposited on inoculated sweet pepper plants (χ^2^ = 1.40, *df* = 2, *P* = 0.495; Fig 3). Overall, hind tibia lengths of wasps were similar across the treatments (*B*. *bassiana*-inoculated plants = 377.17 ± 4.41 μm; *T*. *harzianum*-inoculated plants = 389.98 ± 3.22 μm; non-inoculated control plants = 381.38 ± 2.99 μm). Likewise, the proportion of the egg mass that was parasitized did not differ between the treatments (χ ^2^ = 0.25, *df* = 2, *P* = 0.882; S2 Fig) (*B*. *bassiana*-inoculated plants = 93 ± 5.7%; *T*. *harzianum*-inoculated plants = 97 ± 1.6%; non-inoculated control plants = 94 ± 4.8%).

## Discussion

Plants respond to egg deposition by herbivores by activating a cascade of direct and indirect defence mechanisms [68]. The latter involve the recruitment of the natural enemies of the attacking herbivores through the release of induced plant volatiles [21, 69]. Although recruitment of *T*. *basalis* upon *N*. *viridula* oviposition has been documented in crop species like broad bean and French bean [55], our findings indicate that *T*. *basalis* is also attracted following stink bug egg deposition on sweet pepper. This implies that the attraction of *T*. *basalis* to plants infested with *N*. *viridula* eggs extends across various plant families, indicating a more widespread phenomenon. Further, our study provides clear evidence that the plant-beneficial fungi *T*. *harzianum* T22 and *B*. *bassiana* ARSEF 3097 affect egg parasitoid behaviour differently.

In Y-tube olfactometer bioassays, female wasps exhibited enhanced attraction to plants inoculated with *T*. *harzianum* T22 compared to non-inoculated plants when subjected to *N*. *viridula* egg-laying. This is in line with recent investigations suggesting that *T*. *harzianum* T22 changes the composition of oviposition-induced plant volatiles and enhances the attraction of *T*. *basalis* towards tomato plants induced by *N*. *viridula* egg deposition (Alınç, T, unpublished results). Likewise, the aphid parasitoid *Aphidius ervi* (Haliday) (Hymenoptera: Braconidae) exhibited a stronger preference for aphid-infested tomato plants inoculated with *T*. *harzianum* T22 compared to non-inoculated plants [70]. The authors proposed that the behavioural changes of *A*. *ervi* are due to the upregulation of genes involved in the biosynthesis of terpenoids and salicylate [34, 70]. In contrast, our results revealed no quantitative or qualitative changes in either the VOC blend as a whole or any of the individual compounds emitted by sweet pepper plants inoculated with *T*. *harzianum* compared to non-inoculated plants. However, it has to be noted that, in our study, not only a different plant species was used, but also plants were grown in non-sterile potting mix, in contrast to the aforementioned studies that utilized sterile soil, promoting fungal colonization after inoculation or reducing microbial activity on control plants. As of now, it remains unclear whether soil sterilization may have led to more pronounced differences in VOC profiles between fungus-inoculated and control plants. Similarly to our results, an earlier study, which revealed a greater attraction of the natural enemy *Macrolophus pygmaeus* (Rambur) (Hemiptera: Miridae) to *Trichoderma*-inoculated herbivore-infested plants compared to non-inoculated ones, did not identify a distinct mechanistic foundation for the observed choice pattern through the analysis of VOCs and gene transcription either [28].

When plants were exposed to *N*. *viridula* egg deposition, *B*. *bassiana* inoculation reduced wasp attraction, in contrast to the results obtained for *T*. *harzianum*. A similar negative effect has also been reported for the plant-growth-promoting rhizobacterium *Pseudomonas fluorescens* WCS417r, which decreased the attraction of the parasitoid *Diaeretiella rapae* (M’Intosh) (Hymenoptera: Braconidae) towards *Myzus persicae* (Sulzer) (Hemiptera: Aphididae) infested plants [32]. The authors hypothesized that this was due to significant differences in the emissions of a small number of volatile compounds that they recorded. Our multivariate analysis showed that *B*. *bassiana*-inoculated plants, overall, did not emit significantly different volatile profiles than non-inoculated plants in response to *N*. *viridula* egg deposition. Also, statistical analyses at the single compound level failed to highlight any possible compound(s) that could explain the repellent effect of *B*. *bassiana*. Together, our findings thus indicate that fungal inoculation does not always benefit the plant, at least from an indirect defensive perspective. Indeed, in line with previous research, our study confirms that the outcome of plant-mediated interactions between plant-beneficial fungi and parasitoids depends on a multifaceted interplay involving the herbivore and parasitoid species, as well as the plant and microbial species (or strain) [12, 27, 32, 37, 54, 70, 71].

Although several studies have reported beneficial microbe-mediated effects on plant VOC emissions [17, 72, 73], in some cases the differences are only marginally significant [28], or the VOC analysis cannot explain the behavioural data [74]. Surprisingly, in our study we did not detect any significant differences in the VOC profiles between oviposition-exposed and control plants. Previous research on bean plants showed increased emissions of terpenoids such as linalool, (*E*)-*β*-caryophyllene, (*E*,*E*)-4,8,12-trimethyl-1,3,7,11-tridecatetraene (TMTT), and (3*E*)-4,8-dimethyl-1,3,7-nonatriene (DMNT) in response to feeding and oviposition by *N*. *viridula*, but only (*E*)-*β*-caryophyllene was emitted in higher quantities in feeding-damaged plants with an egg mass compared to only feeding-damaged plants [75]. It is conceivable that, in our case, the causal compounds fell below the detection limit, or were influenced by other factors. Since the setups used for the Y-tube olfactometer assays and headspace sampling were slightly different in our study, and a different set of plants was used in these experiments, this also could have contributed to the discrepancy between our behavioural assays and VOC analysis. Moreover, parasitoids have evolved highly effective mechanisms for detecting subtle variations in the complex blends of VOCs associated with their hosts [41, 76]. Additionally, it is well known that insect behaviour is not always influenced by the volatile blend as a whole or the presence and abundance of specific compounds in the blend, but rather depends on the level and ratio of the different compounds [18, 41, 77, 78]. It must also be noted that minor constituents of the VOC blend are often highly important in natural enemy behaviour, in particular given the highly sensitive olfactory receptor neurons in insect antennae [79]. This suggests that the olfactometer assays might be more indicative of the parasitoid’s fine-tuned perception of specific cues that extend beyond the sensitivity range of the analytical tools used. To better understand the underlying mechanisms explaining the differential recruitment of the parasitoids observed in this study, future studies could apply gas chromatography with electroantennographic detection (GC-EAD) to assess the responses of *T*. *basalis* towards the odours from the differently treated plants. While techniques like GC-MS allow to assess the composition of VOC blends, GC-EAD is suitable to identify biologically active compounds [80, 81].

While we observed contrasting effects of plant-beneficial fungi on the attraction of *T*. *basalis*, the performance of the wasps was not affected when emerging from host eggs laid on non-inoculated *versus* fungal-inoculated plants. There is increasing evidence that plant-associated fungi may affect plant traits with cascading effects on the second and third trophic levels [14, 76, 82]. Given that *T*. *harzianum* T22 has been demonstrated to directly influence *N*. *viridula* performance through the upregulation of jasmonic acid marker genes [10], it is plausible that plant-associated microbes affect host quality and parasitoid performance. However, fitness-related traits of *T*. *basalis*, measured in terms of body size, were not affected by fungal treatments, indicating that fungal inoculation did not affect plant-mediated host quality. Thus, plant-beneficial fungi like *T*. *harzianum* T22 may enhance egg parasitoid fitness primarily by enhancing the attraction of *T*. *basalis* which, in turn, leads to higher host discovery rates. However, once host eggs have been located and parasitized, there are no apparent effects of *T*. *harzianum* T22 on fitness-related traits of the emerging wasps. Nevertheless, it would be interesting in future studies to investigate the impact on wasps emerging from eggs laid by stink bugs that had been feeding on inoculated plants for a larger portion of their life cycle as well.

This study has shown that *T*. *basalis* responds to and is attracted to sweet pepper plants bearing an *N*. *viridula* egg mass. Further, our results showed that plant-beneficial fungi can affect the recruitment of egg parasitoids, but these effects strongly depend on the fungal species. While *T*. *harzianum* T22 enhanced attraction of *T*. *basalis* following egg deposition, *B*. *bassiana* ARSEF 3097 had the opposite effect. In contrast, fitness-related traits of the wasps, determined by the hind tibia length, were not altered by either of the two fungi, indicating that fungal inoculation did not indirectly affect host quality. Altogether, these results suggest that plant inoculation with *T*. *harzianum* T22 can be used to enhance the attraction of egg parasitoids, which could be a promising strategy in manipulating early plant responses against pest insects and improving sustainable crop protection. Further investigations, however, are required to study its effectiveness under field conditions. From a more fundamental point of view, our findings underscore the importance of considering the role of microbes in unravelling the complex interactions between plants, herbivores and their associated egg parasitoids.

## Supporting information

S1 Fig Heatmap of the VOC composition of the headspace of differently treated sweet pepper plants. Plants inoculated with *Beauveria bassiana* ARSEF 3097 or *Trichoderma harzianum* T22, or mock-inoculated with physiological saline solution, were uninfested or subjected to *Nezara viridula* feeding or oviposition. For all tentatively identified compounds, the average peak height is represented as the calculated Z-Score. Compounds are grouped by class, and within the class they are arranged in ascending order of their retention time.
(DOCX)

S2 Fig Proportion of the *Nezara viridula* egg masses deposited on plants inoculated with *Beauveria bassiana* ARSEF 3097 (blue) or *Trichoderma harzianum* T22 (red) or mock-inoculated with physiological water (green) parasitized by *Trissolcus basalis*. The diamond represents the average proportion, while the error bars indicate the standard error.
(DOCX)

S1 Table Volatile compounds (tentative identification) detected and measured in the headspace of differently treated sweet pepper plants. Plants were uninfested or subjected to *Nezara viridula* feeding (F) or oviposition (O), and inoculated with *Beauveria bassiana* ARSEF 3097 (Bb) or *Trichoderma harzianum* T22 (Th) or mock-inoculated with physiological saline solution (Co).
(DOCX)

S2 Table *P* values of univariate significance tests for all putatively identified VOCs, adjusted for multiple testing using the Benjamini-Hochberg correction to control the false discovery rate at 5%. Adjusted *P* values are shown for all combinations tested in the olfactometer bioassays. Plants were uninfested or subjected to *Nezara viridula* feeding (F) or oviposition (O), and inoculated with *Beauveria bassiana* ARSEF 3097 (Bb) or *Trichoderma harzianum* T22 (Th) or mock-inoculated with physiological water (Co).
(DOCX)

S1 Data Data behind all results presented in this manuscript. Underlying data for Figs 1–3 and S1–S2 Figs and S1 and S2 Tables.
(XLSX)
