# Supplementary material for: Differential effects of plant-beneficial fungi on the attraction of the egg parasitoid Trissolcus basalis in response to Nezara viridula egg deposition
Source: PLoS One. 2024 May 21;19(5):e0304220. doi: 10.1371/journal.pone.0304220 (PMC11108215; doi:10.1371/journal.pone.0304220)
Supplement: S1 Fig — Plants inoculated with Beauveria bassiana ARSEF 3097 or Trichoderma harzianum T22, or mock-inoculated with physiological saline solution, were uninfested or subjected to Nezara viridula feeding or oviposition. For all tentatively identified compounds, the average peak height is represented as the calculated Z-Score. Compounds are grouped by class, and within the class they are arranged in ascending order of their retention time. (DOCX) [file pone.0304220.s001.docx]

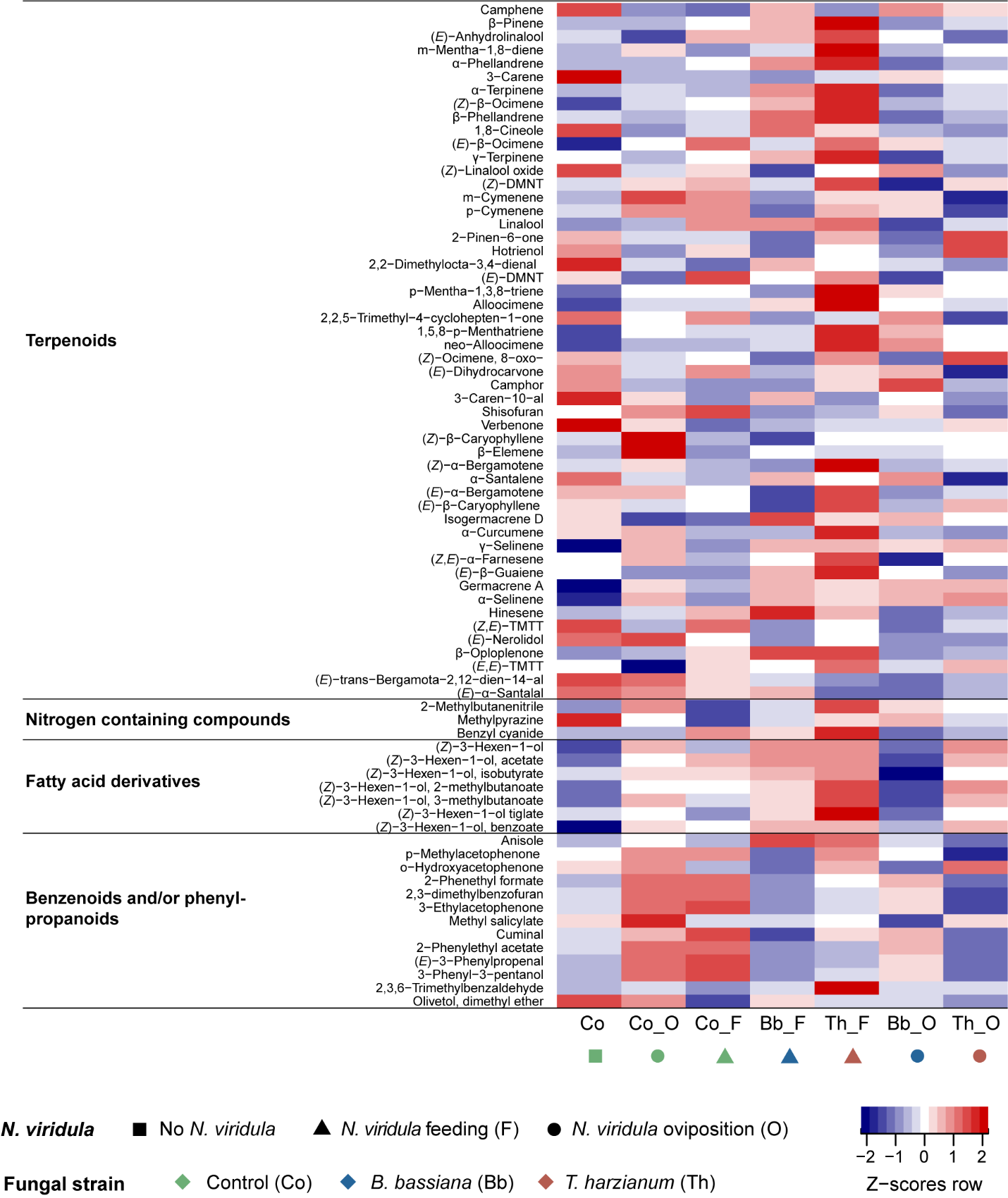


**S1 Fig.** **Heatmap of the VOC composition of the headspace of differently treated sweet pepper plants.** Plants inoculated with *Beauveria bassiana* ARSEF 3097 or *Trichoderma harzianum* T22, or mock-inoculated with physiological saline solution, were uninfested or subjected to *Nezara viridula* feeding or oviposition. For all tentatively identified compounds, the average peak height is represented as the calculated Z-Score. Compounds are grouped by class, and within the class they are arranged in ascending order of their retention time.
