# Supplementary material for: Differential effects of plant-beneficial fungi on the attraction of the egg parasitoid Trissolcus basalis in response to Nezara viridula egg deposition
Source: PLoS One. 2024 May 21;19(5):e0304220. doi: 10.1371/journal.pone.0304220 (PMC11108215; doi:10.1371/journal.pone.0304220)
Supplement: S2 Fig — The diamond represents the average proportion, while the error bars indicate the standard error. (DOCX) [file pone.0304220.s002.docx]

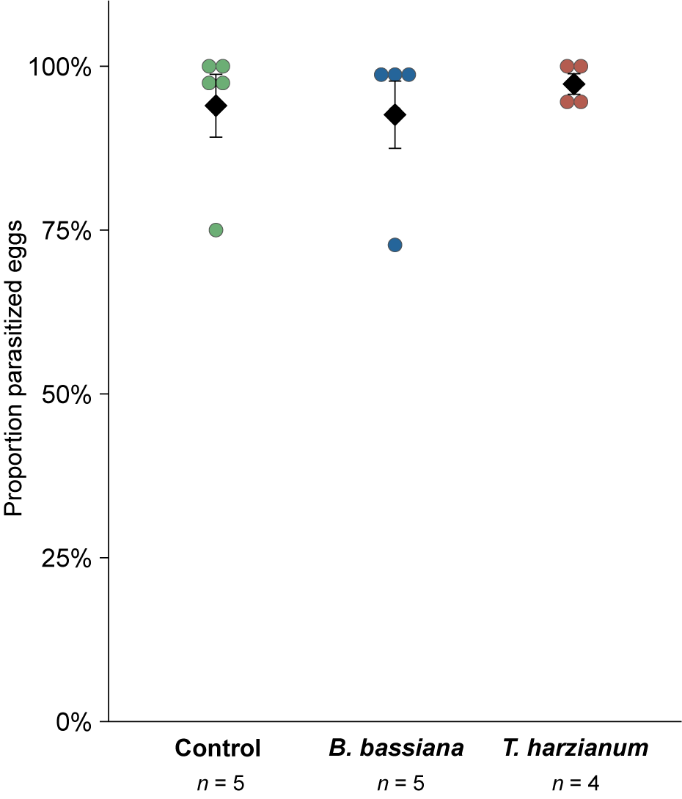


**S2 Fig.** **Proportion of these *Nezara viridula* egg masses deposited on plants inoculated with *Beauveria bassiana* ARSEF 3097 (blue) or *Trichoderma harzianum* T22 (red) or mock-inoculated with physiological water (green) parasitized by *Trissolcus basalis*.** The diamond represents the average proportion, while the error bars indicate the standard error.
