# Supplementary material for: Differential effects of plant-beneficial fungi on the attraction of the egg parasitoid Trissolcus basalis in response to Nezara viridula egg deposition
Source: PLoS One. 2024 May 21;19(5):e0304220. doi: 10.1371/journal.pone.0304220 (PMC11108215; doi:10.1371/journal.pone.0304220)
Supplement: S1 Table — Plants were uninfested or subjected to Nezara viridula feeding (F) or oviposition (O), and inoculated with Beauveria bassiana ARSEF 3097 (Bb) or Trichoderma harzianum T22 (Th) or mock-inoculated with physiological saline solution (Co). (DOCX) [file pone.0304220.s003.docx]

**S1 Table.** **Volatile compounds^a^ (tentative identification) detected and measured in the headspace of differently treated sweet pepper plants.** Plants were uninfested or subjected to *Nezara viridula* feeding (F) or oviposition (O), and inoculated with *Beauveria bassiana* ARSEF 3097 (Bb) or *Trichoderma harzianum* T22 (Th) or mock-inoculated with physiological saline solution (Co).

| **Compound name and class** | | **RT^b^  (min)** | **ERI^c^** | **LRI^d^** | **Co (*n* = 9)** | **Co_O  (*n* = 9)** | **Co_F  (*n* = 9)** | **Bb_F  (*n* = 9)** | **Th_F  (*n* = 9)** | **Bb_O  (*n* = 10)** | **Th_O  (*n* = 10)** | |  |
| --- | --- | --- | --- | --- | --- | --- | --- | --- | --- | --- | --- | --- | --- |
| **Terpenoids** | | | | | | | | | | | |  |  |
|  | Camphene | 12.55 | 963 | 962 | 1046.8 ± 584.8 | 442.3 ± 180.1 | 383.8 ± 149.1 | 717.1 ± 269.9 | 402.8 ± 115.7 | 809.8 ± 282.6 | 636.1 ± 193.4 | |  |
|  | β-Pinene | 13.32 | 990 | 989 | 50.9 ± 8.8 | 52.0 ± 11.5 | 73.5 ± 20.4 | 123.3 ± 26.1 | 276.8 ± 125.2 | 42.1 ± 11.1 | 64.5 ± 14.4 | |  |
|  | (*E*)-Anhydrolinalool | 13.46 | 995 | 993 | 27.6 ± 10.2 | 17.3 ± 7.0 | 39.3 ± 18.1 | 38.4 ± 16.8 | 51.7 ± 19.0 | 28.9 ± 7.1 | 18.2 ± 5.4 | |  |
|  | m-Mentha-1,8-diene | 14.00 | 1014 | 1009 | 14.3 ± 4.3 | 26.0 ± 10.9 | 10.1 ± 3.1 | 17.0 ± 5.4 | 107.6 ± 76.8 | 12.9 ± 3.2 | 21.8 ± 9.9 | |  |
|  | α-Phellandrene | 14.06 | 1016 | 1017 | 4.0 ± 0.8 | 4.2 ± 1.1 | 5.5 ± 2.3 | 10.7 ± 3.5 | 19.8 ± 9.6 | 3.0 ± 0.7 | 4.4 ± 1.1 | |  |
|  | 3-Carene | 14.15 | 1019 | 1019 | 173.0 ± 53.5 | 84.1 ± 30.4 | 82.8 ± 27.7 | 73.7 ± 12.9 | 89.3 ± 20.3 | 107.5 ± 18.7 | 102.0 ± 34.1 | |  |
|  | α-Terpinene | 14.36 | 1026 | 1025 | 52.7 ± 5.6 | 63.5 ± 15.3 | 55.1 ± 18.6 | 106.4 ± 30.4 | 178.0 ± 93.8 | 40.8 ± 8.9 | 59.7 ± 13.9 | |  |
|  | (*Z*)-β-Ocimene | 14.64 | 1035 | 1037 | 73.4 ± 22.3 | 193.9 ± 69.3 | 230.4 ± 106.1 | 394.8 ± 124.9 | 1267.4 ± 615.0 | 167.6 ± 57.3 | 212.7 ± 61.5 | |  |
|  | β-Phellandrene | 14.85 | 1043 | 1044 | 15.7 ± 2.5 | 14.6 ± 2.5 | 17.1 ± 4.5 | 28.7 ± 8.6 | 38.1 ± 16.7 | 11.8 ± 2.3 | 14.3 ± 3.5 | |  |
|  | 1,8-Cineole | 14.91 | 1045 | 1044 | 72.2 ± 8.4 | 48.8 ± 8.5 | 55.4 ± 10.2 | 69.3 ± 13.2 | 59.4 ± 9.9 | 51.2 ± 8.0 | 48.4 ± 7.1 | |  |
|  | (*E*)-β-Ocimene | 15.00 | 1048 | 1048 | 8.5 ± 4.7 | 57.5 ± 23.2 | 201.5 ± 168.7 | 46.0 ± 20.4 | 214.2 ± 101.8 | 84.3 ± 44.6 | 53.4 ± 21.8 | |  |
|  | γ-Terpinene | 15.54 | 1066 | 1065 | 15.0 ± 2.4 | 12.8 ± 2.9 | 14.6 ± 3.3 | 18.8 ± 4.0 | 28.7 ± 11.3 | 9.6 ± 1.7 | 13.6 ± 3.2 | |  |
|  | (*Z*)-Linalool oxide | 15.92 | 1079 | 1079 | 49.8 ± 14.0 | 29.0 ± 14.3 | 36.7 ± 11.1 | 20.9 ± 3.3 | 33.0 ± 7.7 | 41.6 ± 5.8 | 24.2 ± 4.6 | |  |
|  | (*Z*)-DMNT^e^ | 16.40 | 1095 | 1094 | 272.3 ± 161.9 | 339.0 ± 171.1 | 361.9 ± 147.6 | 292.8 ± 115.9 | 478.6 ± 208.9 | 166.8 ± 65.5 | 354.3 ± 212.1 | |  |
|  | m-Cymenene | 16.40 | 1095 | 1088 | 30.2 ± 4.5 | 57.1 ± 37.2 | 46.3 ± 21.7 | 27.2 ± 5.3 | 40.5 ± 10.7 | 39.4 ± 9.1 | 19.9 ± 2.3 | |  |
|  | p-Cymenene | 16.54 | 1100 | 1103 | 255.3 ± 29.1 | 422.8 ± 214.3 | 430.4 ± 205.5 | 185.9 ± 29.5 | 376.6 ± 102.5 | 339.4 ± 76.5 | 176.0 ± 21.6 | |  |
|  | Linalool | 16.59 | 1101 | 1101 | 74.3 ± 30.6 | 115.7 ± 54.8 | 478.1 ± 236.5 | 420.3 ± 125.3 | 562.3 ± 204.5 | 48.0 ± 10.4 | 136.1 ± 70.7 | |  |
|  | 2-Pinen-6-one | 16.69 | 1105 | 1106 | 342.0 ± 258.2 | 228.4 ± 140.2 | 236.0 ± 114.9 | 152.0 ± 54.0 | 332.8 ± 164.8 | 156.9 ± 80.2 | 459.7 ± 334.9 | |  |
|  | Hotrienol | 16.86 | 1112 | 1110 | 93.8 ± 71.2 | 24.9 ± 10.2 | 60.7 ± 39.2 | 21.1 ± 5.8 | 47.5 ± 18.0 | 21.9 ± 3.7 | 133.1 ± 101.6 | |  |
|  | 2,2-Dimethylocta-3,4-dienal | 16.87 | 1112 | 1116 | 224.7 ± 48.2 | 126.1 ± 23.6 | 97.7 ± 32.5 | 157.5 ± 29.5 | 139.0 ± 27.0 | 126.0 ± 29.5 | 101.5 ± 24.7 | |  |
|  | (*E*)-DMNT^e^ | 16.98 | 1116 | 1115 | 667.8 ± 323.4 | 411.3 ± 169.1 | 980.8 ± 285.8 | 618.4 ± 192.0 | 782.8 ± 200.2 | 355.7 ± 113.8 | 605.0 ± 195.0 | |  |
|  | p-Mentha-1,3,8-triene | 17.30 | 1128 | 1119 | 9.2 ± 1.3 | 47.6 ± 18.4 | 35.9 ± 17.6 | 14.9 ± 9.7 | 442.9 ± 285.9 | 60.0 ± 29.6 | 36.1 ± 19.2 | |  |
|  | Alloocimene | 17.43 | 1133 | 1232 | 9.7 ± 3.6 | 42.8 ± 17.5 | 36.6 ± 18.7 | 64.4 ± 24.3 | 510.2 ± 312.1 | 52.4 ± 22.9 | 42.2 ± 19.4 | |  |
|  | 2,2,5-Trimethyl-4-cyclohepten-1-one | 17.56 | 1138 | 1149 | 35.4 ± 7.3 | 28.7 ± 12.2 | 34.0 ± 10.9 | 23.7 ± 3.5 | 27.2 ± 5.3 | 34.3 ± 4.4 | 21.1 ± 3.6 | |  |
|  | 1,5,8-p-Menthatriene | 17.59 | 1139 | 1139 | 7.7 ± 2.6 | 48.2 ± 20.1 | 37.0 ± 18.1 | 34.5 ± 15.1 | 529.8 ± 337.2 | 94.3 ± 46.6 | 46.6 ± 21.0 | |  |
|  | neo-Alloocimene | 17.83 | 1148 | 1144 | 3.5 ± 1.2 | 17.6 ± 6.8 | 16.9 ± 7.8 | 25.6 ± 9.4 | 539.1 ± 344.8 | 138.3 ± 82.7 | 42.6 ± 22.9 | |  |
|  | (*Z*)-Ocimene, 8-oxo- | 17.94 | 1152 | NA | 176.2 ± 124.7 | 102.5 ± 64.4 | 122.6 ± 56.5 | 68.6 ± 23.6 | 194.2 ± 97.6 | 61.1 ± 32.1 | 270.3 ± 207.2 | |  |
|  | (*E*)-Dihydrocarvone | 18.01 | 1154 | 1180 | 18.4 ± 3.1 | 14.5 ± 4.9 | 17.6 ± 4.9 | 14.0 ± 2.3 | 16.3 ± 3.3 | 17.5 ± 1.9 | 11.4 ± 1.8 | |  |
|  | Camphor | 18.40 | 1169 | 1171 | 407.0 ± 66.0 | 288.5 ± 61.4 | 270.0 ± 47.8 | 276.4 ± 41.3 | 360.5 ± 65.3 | 462.3 ± 56.9 | 291.0 ± 47.9 | |  |
|  | 3-Caren-10-al | 18.51 | 1173 | NA | 8.2 ± 1.3 | 6.1 ± 1.4 | 4.8 ± 1.4 | 6.7 ± 1.5 | 4.9 ± 0.9 | 5.8 ± 0.9 | 4.7 ± 1.0 | |  |
|  | Shisofuran | 18.65 | 1178 | 1198 | 20.7 ± 2.3 | 29.2 ± 14.2 | 34.0 ± 18.1 | 16.3 ± 3.5 | 18.2 ± 5.7 | 24.3 ± 4.8 | 14.5 ± 2.9 | |  |
|  | Verbenone | 19.80 | 1227 | 1226 | 212.6 ± 52.8 | 99.6 ± 40.2 | 47.8 ± 26.0 | 62.5 ± 17.7 | 74.4 ± 28.0 | 73.5 ± 27.0 | 91.7 ± 47.2 | |  |
|  | (*Z*)-β-Caryophyllene | 23.07 | 1401 | 1409 | 53.0 ± 17.0 | 205.7 ± 131.4 | 44.9 ± 13.0 | 26.7 ± 11.7 | 65.6 ± 24.9 | 55.4 ± 12.8 | 62.3 ± 13.4 | |  |
|  | β-Elemene | 23.20 | 1410 | 1416 | 46.2 ± 17.5 | 168.0 ± 89.9 | 44.4 ± 11.7 | 59.9 ± 25.4 | 56.5 ± 17.7 | 57.6 ± 13.4 | 59.7 ± 13.4 | |  |
|  | (*Z*)-α-Bergamotene | 23.54 | 1432 | 1413 | 34.2 ± 7.6 | 43.7 ± 11.8 | 30.2 ± 7.7 | 24.4 ± 3.8 | 112.2 ± 73.5 | 27.2 ± 7.2 | 34.4 ± 9.0 | |  |
|  | α-Santalene | 23.65 | 1440 | 1440 | 180.8 ± 19.2 | 139.7 ± 14.7 | 132.9 ± 17.9 | 161.8 ± 18.8 | 144.6 ± 20.6 | 169.4 ± 29.4 | 110.9 ± 15.9 | |  |
|  | (*E*)-α-Bergamotene | 23.85 | 1453 | 1455 | 1282.4 ± 332.8 | 1236.2 ± 207.0 | 1122.3 ± 202.2 | 878.6 ± 90.1 | 1464.2 ± 510.3 | 986.5 ± 148.5 | 1091.6 ± 196.3 | |  |
|  | (E)-β-Caryophyllene | 23.93 | 1458 | 1455 | 294.4 ± 125.2 | 233.0 ± 109.8 | 278.5 ± 87.6 | 151.1 ± 19.5 | 463.1 ± 221.6 | 209.8 ± 53.5 | 343.9 ± 79.6 | |  |
|  | Isogermacrene D | 24.04 | 1466 | 1452 | 9.4 ± 1.1 | 6.5 ± 1.4 | 7.2 ± 1.2 | 11.2 ± 1.7 | 9.2 ± 2.1 | 9.6 ± 1.1 | 8.7 ± 1.1 | |  |
|  | α-Curcumene | 24.51 | 1497 | 1493 | 105.8 ± 23.9 | 117.2 ± 31.1 | 84.4 ± 17.3 | 86.4 ± 7.8 | 156.6 ± 61.8 | 85.7 ± 13.1 | 82.8 ± 12.4 | |  |
|  | γ-Selinene | 24.59 | 1502 | 1509 | 9.8 ± 3.2 | 28.9 ± 11.4 | 16.1 ± 3.8 | 27.7 ± 10.0 | 27.6 ± 9.0 | 26.3 ± 10.6 | 28.8 ± 7.8 | |  |
|  | (*Z*,*E*)-α-Farnesene | 24.69 | 1510 | 1505 | 79.5 ± 31.0 | 97.7 ± 45.2 | 65.7 ± 21.4 | 76.1 ± 15.5 | 136.0 ± 51.4 | 42.4 ± 11.3 | 82.6 ± 19.3 | |  |
|  | (*E*)-β-Guaiene | 24.84 | 1521 | 1503 | 32.9 ± 6.0 | 29.2 ± 6.9 | 29.4 ± 4.7 | 35.7 ± 5.1 | 42.3 ± 10.4 | 32.6 ± 9.8 | 29.2 ± 7.4 | |  |
|  | Germacrene A | 24.93 | 1527 | 1517 | 45.1 ± 14.8 | 140.7 ± 53.4 | 83.6 ± 19.2 | 162.3 ± 51.5 | 143.5 ± 45.9 | 172.9 ± 76.2 | 172.4 ± 50.3 | |  |
|  | α-Selinene | 25.01 | 1533 | 1534 | 122.3 ± 37.9 | 524.8 ± 212.3 | 213.8 ± 62.6 | 496.0 ± 196.4 | 406.5 ± 146.4 | 474.5 ± 169.2 | 551.3 ± 169.3 | |  |
|  | Hinesene | 25.06 | 1537 | 1495 | 43.8 ± 16.4 | 55.7 ± 9.3 | 91.7 ± 61.2 | 176.1 ± 75.3 | 90.0 ± 23.4 | 30.4 ± 9.0 | 41.9 ± 9.4 | |  |
|  | (*Z*,*E*)-TMTT^f^ | 25.41 | 1563 | 1560 | 153.7 ± 89.0 | 63.5 ± 29.3 | 136.0 ± 90.0 | 57.1 ± 26.3 | 82.4 ± 36.0 | 48.4 ± 16.8 | 71.2 ± 19.0 | |  |
|  | (*E*)-Nerolidol | 25.51 | 1570 | 1570 | 228.2 ± 117.8 | 275.5 ± 144.5 | 86.3 ± 29.1 | 40.5 ± 23.6 | 84.3 ± 39.7 | 35.6 ± 16.1 | 39.5 ± 17.6 | |  |
|  | β-Oploplenone | 25.56 | 1574 | 1575 | 18.9 ± 2.3 | 22.7 ± 7.0 | 34.7 ± 14.1 | 67.9 ± 28.0 | 65.6 ± 25.0 | 18.1 ± 3.4 | 21.4 ± 3.1 | |  |
|  | (*E*,*E*)-TMTT^f^ | 25.67 | 1582 | 1581 | 287.4 ± 126.3 | 93.3 ± 28.5 | 327.6 ± 118.0 | 275.3 ± 141.4 | 538.9 ± 265.5 | 212.9 ± 77.7 | 339.7 ± 108.3 | |  |
|  | (*E*)-trans-Bergamota-2,12-dien-14-al | 25.98 | 1605 | 1622 | 342.1 ± 226.4 | 280.5 ± 188.9 | 146.6 ± 123.6 | 107.5 ± 56.6 | 68.2 ± 26.8 | 48.0 ± 16.1 | 72.3 ± 17.0 | |  |
|  | (*E*)-α-Santalal | 26.34 | 1634 | 1679 | 188.0 ± 121.4 | 161.2 ± 117.6 | 79.5 ± 64.8 | 110.8 ± 77.6 | 25.8 ± 7.4 | 25.8 ± 9.7 | 39.6 ± 11.5 | |  |
| **Nitrogen containing compounds** | | | | | | | | | | | | | |
|  | 2-Methylbutanenitrile | 6.71 | 729 | 731 | 85.6 ± 29.5 | 660.0 ± 327.4 | 56.6 ± 20.0 | 194.8 ± 88.4 | 1644.4 ± 754.9 | 401.5 ± 216.0 | 268.1 ± 223.7 | |  |
|  | Methylpyrazine | 8.94 | 826 | 826 | 339.7 ± 101.7 | 160.2 ± 31.3 | 83.8 ± 33.3 | 137.3 ± 37.5 | 183.9 ± 54.4 | 197.8 ± 69.0 | 136.3 ± 48.1 | |  |
|  | Benzyl cyanide | 17.89 | 1150 | 1150 | 37.8 ± 5.2 | 37.6 ± 7.5 | 197.6 ± 96.5 | 96.0 ± 26.1 | 734.3 ± 413.7 | 22.9 ± 4.9 | 45.9 ± 19.9 | |  |
| **Fatty acid derivatives** | | | | | | | | | | | | | |
|  | (*Z*)-3-Hexen-1-ol | 9.61 | 853 | 859 | 344.3 ± 122.4 | 1693.3 ± 891.3 | 582.8 ± 303.6 | 2407.1 ± 635.5 | 2446.5 ± 1024.1 | 421.7 ± 214.9 | 2038.5 ± 968.3 | |  |
|  | (*Z*)-3-Hexen-1-ol, acetate | 13.65 | 1002 | 1005 | 105.5 ± 44.2 | 582.2 ± 275.5 | 1299.6 ± 870.3 | 1475.1 ± 541.9 | 1842.8 ± 828.9 | 81.5 ± 28.6 | 1058.0 ± 596.8 | |  |
|  | (*Z*)-3-Hexen-1-ol, isobutyrate | 17.71 | 1143 | 1142 | 23.3 ± 19.0 | 102.0 ± 65.4 | 126.8 ± 80.6 | 148.4 ± 105.1 | 552.5 ± 275.1 | 0.2 ± 0.0 | 44.4 ± 23.9 | |  |
|  | (*Z*)-3-Hexen-1-ol, 2-methylbutanoate | 19.91 | 1233 | 1230 | 28.3 ± 7.1 | 120.7 ± 81.8 | 133.6 ± 85.8 | 267.6 ± 140.7 | 1222.4 ± 702.1 | 21.3 ± 5.7 | 461.6 ± 287.0 | |  |
|  | (*Z*)-3-Hexen-1-ol, 3-methylbutanoate | 20.00 | 1237 | 1237 | 15.3 ± 8.2 | 130.2 ± 100.2 | 50.4 ± 19.9 | 106.2 ± 62.3 | 481.5 ± 301.9 | 8.4 ± 3.7 | 170.9 ± 104.5 | |  |
|  | (*Z*)-3-Hexen-1-ol tiglate | 21.77 | 1326 | 1325 | 6.4 ± 3.9 | 14.8 ± 13.2 | 2.7 ± 1.1 | 19.9 ± 13.4 | 277.8 ± 259.7 | 2.1 ± 0.3 | 12.5 ± 7.0 | |  |
|  | (*Z*)-3-Hexen-1-ol, benzoate | 25.80 | 1591 | 1588 | 0.2 ± 0.0 | 149.2 ± 81.0 | 70.1 ± 30.2 | 478.6 ± 229.1 | 461.8 ± 217.5 | 12.3 ± 8.1 | 472.4 ± 437.5 | |  |
| **Benzenoids and/or phenylpropanoids** | | | | | | | | | | | | | |
|  | Anisole | 11.41 | 923 | 923 | 83.6 ± 10.0 | 99.9 ± 23.9 | 84.0 ± 17.7 | 148.0 ± 35.3 | 134.0 ± 24.4 | 90.5 ± 8.3 | 73.5 ± 10.2 | |  |
|  | p-Methylacetophenone | 17.46 | 1134 | 1156 | 248.8 ± 39.1 | 308.9 ± 137.6 | 305.8 ± 139.2 | 176.4 ± 22.8 | 326.9 ± 93.8 | 250.8 ± 52.6 | 146.1 ± 16.2 | |  |
|  | o-Hydroxyacetophenone | 18.61 | 1177 | 1167 | 122.4 ± 51.7 | 163.4 ± 99.8 | 81.9 ± 20.5 | 66.3 ± 13.9 | 141.3 ± 53.5 | 66.6 ± 13.4 | 182.0 ± 120.7 | |  |
|  | 2-Phenethyl formate | 18.77 | 1183 | 1185 | 198.1 ± 21.0 | 496.2 ± 327.8 | 492.1 ± 342.4 | 153.3 ± 19.9 | 261.6 ± 89.8 | 345.5 ± 111.8 | 130.0 ± 13.1 | |  |
|  | 2,3-dimethylbenzofuran | 19.26 | 1201 | 1222 | 40.3 ± 7.5 | 118.9 ± 87.8 | 114.4 ± 87.0 | 25.9 ± 9.3 | 42.1 ± 16.8 | 55.8 ± 22.8 | 18.3 ± 5.4 | |  |
|  | 3-Ethylacetophenone | 19.38 | 1207 | 1186 | 124.5 ± 16.2 | 214.0 ± 134.2 | 246.9 ± 161.7 | 92.6 ± 13.3 | 124.2 ± 27.5 | 143.2 ± 47.9 | 73.4 ± 8.5 | |  |
|  | Methyl salicylate | 19.39 | 1208 | 1208 | 60.9 ± 23.9 | 211.7 ± 111.3 | 35.3 ± 23.8 | 32.6 ± 15.3 | 41.1 ± 29.9 | 13.2 ± 5.3 | 56.3 ± 32.4 | |  |
|  | Cuminal | 19.64 | 1220 | 1220 | 8.4 ± 1.8 | 12.7 ± 8.4 | 16.4 ± 8.9 | 6.0 ± 0.8 | 11.1 ± 2.8 | 12.1 ± 2.4 | 6.3 ± 1.1 | |  |
|  | 2-Phenylethyl acetate | 20.05 | 1239 | 1235 | 94.0 ± 14.8 | 235.1 ± 164.9 | 236.6 ± 180.0 | 61.6 ± 12.2 | 77.3 ± 20.3 | 151.5 ± 51.9 | 54.2 ± 6.2 | |  |
|  | (*E*)-3-Phenylpropenal | 20.21 | 1247 | 1253 | 70.3 ± 9.8 | 203.8 ± 149.2 | 220.9 ± 170.3 | 57.7 ± 11.8 | 72.2 ± 18.7 | 120.6 ± 45.7 | 45.0 ± 4.8 | |  |
|  | 3-Phenyl-3-pentanol | 20.33 | 1253 | NA | 53.0 ± 6.9 | 145.5 ± 103.4 | 152.9 ± 112.5 | 46.2 ± 8.4 | 58.6 ± 16.1 | 85.4 ± 31.5 | 35.4 ± 3.6 | |  |
|  | 2,3,6-Trimethylbenzaldehyde | 21.98 | 1339 | 1339 | 11.3 ± 2.8 | 16.4 ± 5.8 | 9.9 ± 1.8 | 17.0 ± 5.4 | 130.2 ± 80.6 | 16.5 ± 6.0 | 16.2 ± 5.3 | |  |
|  | Olivetol, dimethyl ether | 26.46 | 1644 | 1644 | 839.2 ± 171.3 | 784.6 ± 107.0 | 637.3 ± 125.4 | 744.6 ± 159.7 | 713.7 ± 168.6 | 715.4 ± 149.4 | 673.9 ± 127.8 | |  |

^a^ Volatile emissions are presented as average peak heights ± SE /g fresh weight of foliage, divided by 10^2^. The number of biological replicates is given in parentheses.

^b^ RT = retention time (in minutes) of compounds.

^c^ ERI = experimentally obtained retention indices.

^d^ LRI = retention indices obtained from literature.

^e^ DMNT = 4,8-Dimethylnona-1,3,7-triene.

^f^ TMTT = 4,8,12-Trimethyl-1,3,7,11-tridecatetraene
