# Supplementary material for: Differential effects of plant-beneficial fungi on the attraction of the egg parasitoid Trissolcus basalis in response to Nezara viridula egg deposition
Source: PLoS One. 2024 May 21;19(5):e0304220. doi: 10.1371/journal.pone.0304220 (PMC11108215; doi:10.1371/journal.pone.0304220)
Supplement: S2 Table — Adjusted P values are shown for all combinations tested in the olfactometer bioassays. Plants were uninfested or subjected to Nezara viridula feeding (F) or oviposition (O), and inoculated with Beauveria bassiana ARSEF 3097 (Bb) or Trichoderma harzianum T22 (Th) or mock-inoculated with physiological water (Co). (DOCX) [file pone.0304220.s004.docx]

**S2 Table.** ***P* values of univariate significance tests for all putatively identified VOCs^a^, adjusted for multiple testing using the Benjamini-Hochberg correction to control the false discovery rate at 5%.** Adjusted *P* values are shown for all combinations tested in the olfactometer bioassays. Plants were uninfested or subjected to *Nezara viridula* feeding (F) or oviposition (O), and inoculated with *Beauveria bassiana* ARSEF 3097 (Bb) or *Trichoderma harzianum* T22 (Th) or mock-inoculated with physiological water (Co).

| **Compound name and class** | | **Co**  ***versus***  **Co_O** | **Co**  ***versus***  **Co_F** | **Co_F**  ***versus***  **Bb_F** | **Co_F**  ***versus***  **Th_F** | **Co_O**  ***versus***  **Bb_O** | **Co_O**  ***versus***  **Th_O** |
| --- | --- | --- | --- | --- | --- | --- | --- |
| **Terpenoids** | |  |  |  |  |  |  |
|  | Camphene | 0.832 | 0.746 | 0.672 | 0.89 | 1 | 0.994 |
|  | β-Pinene | 1 | 0.913 | 0.649 | 0.89 | 1 | 0.994 |
|  | (*E*)-Anhydrolinalool | 0.936 | 0.862 | 0.86 | 0.89 | 1 | 0.994 |
|  | m-Mentha-1,8-diene | 1 | 0.684 | 0.649 | 0.89 | 1 | 0.994 |
|  | α-Phellandrene | 1 | 0.862 | 0.649 | 0.89 | 1 | 0.994 |
|  | 3-Carene | 0.649 | 0.547 | 0.928 | 0.89 | 1 | 0.994 |
|  | α-Terpinene | 1 | 0.746 | 0.649 | 0.89 | 1 | 0.994 |
|  | (*Z*)-β-Ocimene | 0.649 | 0.684 | 0.649 | 0.89 | 1 | 0.994 |
|  | β-Phellandrene | 1 | 0.935 | 0.649 | 0.89 | 1 | 0.994 |
|  | 1,8-Cineole | 0.649 | 0.662 | 0.777 | 0.915 | 1 | 0.994 |
|  | (*E*)-β-Ocimene | 0.649 | 0.547 | 0.993 | 0.89 | 1 | 0.994 |
|  | γ-Terpinene | 0.859 | 0.862 | 0.72 | 0.89 | 1 | 1 |
|  | (*Z*)-Linalool oxide | 0.649 | 0.762 | 0.649 | 0.988 | 1 | 0.994 |
|  | (*Z*)-DMNT^b^ | 0.773 | 0.694 | 0.889 | 0.89 | 1 | 0.994 |
|  | m-Cymenene | 0.649 | 0.862 | 0.86 | 0.988 | 1 | 0.994 |
|  | p-Cymenene | 0.832 | 0.862 | 0.818 | 0.89 | 1 | 0.994 |
|  | Linalool | 1 | 0.547 | 0.873 | 0.901 | 1 | 0.994 |
|  | 2-Pinen-6-one | 0.649 | 0.662 | 0.909 | 0.89 | 1 | 0.994 |
|  | Hotrienol | 0.964 | 0.862 | 0.937 | 0.89 | 1 | 0.994 |
|  | 2,2-Dimethylocta-3,4-dienal | 0.649 | 0.647 | 0.649 | 0.89 | 1 | 0.994 |
|  | (*E*)-DMNT^b^ | 0.832 | 0.684 | 0.968 | 0.89 | 1 | 0.994 |
|  | p-Mentha-1,3,8-triene | 0.865 | 0.982 | 0.781 | 0.929 | 1 | 0.994 |
|  | Alloocimene | 0.649 | 0.694 | 0.649 | 0.89 | 1 | 0.994 |
|  | 2,2,5-Trimethyl-4-cyclohepten-1-one | 0.649 | 0.862 | 0.86 | 0.89 | 1 | 0.994 |
|  | 1,5,8-p-Menthatriene | 0.649 | 0.647 | 0.86 | 0.89 | 1 | 1 |
|  | neo-Alloocimene | 0.649 | 0.547 | 0.649 | 0.89 | 1 | 0.994 |
|  | (*Z*)-Ocimene, 8-oxo- | 1 | 0.856 | 0.993 | 0.89 | 1 | 0.994 |
|  | (*E*)-Dihydrocarvone | 0.649 | 0.862 | 0.873 | 0.988 | 1 | 0.994 |
|  | Camphor | 0.649 | 0.547 | 0.968 | 0.89 | 1 | 0.994 |
|  | 3-Caren-10-al | 0.832 | 0.647 | 0.781 | 0.901 | 1 | 0.994 |
|  | Shisofuran | 0.649 | 0.746 | 0.731 | 0.89 | 1 | 0.994 |
|  | Verbenone | 0.649 | 0.547 | 0.731 | 0.89 | 1 | 0.994 |
|  | (*Z*)-β-Caryophyllene | 1 | 1 | 0.649 | 0.89 | 1 | 0.994 |
|  | β-Elemene | 0.649 | 0.862 | 0.86 | 0.89 | 1 | 0.994 |
|  | (*Z*)-α-Bergamotene | 1 | 0.862 | 0.928 | 0.89 | 1 | 0.994 |
|  | α-Santalene | 0.649 | 0.647 | 0.672 | 0.89 | 1 | 0.994 |
|  | (*E*)-α-Bergamotene | 1 | 0.982 | 0.873 | 0.988 | 1 | 0.994 |
|  | (*E*)-β-Caryophyllene | 1 | 0.862 | 0.781 | 0.915 | 1 | 0.994 |
|  | Isogermacrene D | 0.649 | 0.647 | 0.649 | 0.89 | 1 | 0.994 |
|  | α-Curcumene | 1 | 0.779 | 0.831 | 0.89 | 1 | 0.994 |
|  | γ-Selinene | 0.649 | 0.662 | 0.781 | 0.89 | 1 | 0.994 |
|  | (*Z*,*E*)-α-Farnesene | 1 | 0.991 | 0.86 | 0.89 | 1 | 0.994 |
|  | (*E*)-β-Guaiene | 0.936 | 0.762 | 0.89 | 0.89 | 1 | 0.994 |
|  | Germacrene A | 0.649 | 0.647 | 0.72 | 0.89 | 1 | 0.994 |
|  | α-Selinene | 0.649 | 0.684 | 0.672 | 0.89 | 1 | 0.994 |
|  | Hinesene | 0.649 | 0.913 | 0.649 | 0.89 | 1 | 0.994 |
|  | (*Z*,*E*)-TMTT^c^ | 1 | 0.862 | 0.937 | 1 | 1 | 0.994 |
|  | (*E*)-Nerolidol | 1 | 0.984 | 0.672 | 1 | 1 | 0.994 |
|  | β-Oploplenone | 1 | 0.746 | 0.649 | 0.89 | 1 | 0.994 |
|  | (*E*,*E*)-TMTT^c^ | 1 | 0.746 | 0.89 | 1 | 1 | 0.994 |
|  | (*E*)-trans-Bergamota-2,12-dien-14-al | 0.87 | 0.982 | 0.649 | 0.89 | 1 | 0.994 |
|  | (*E*)-α-Santalal | 1 | 0.862 | 0.672 | 0.89 | 1 | 0.994 |
| **Nitrogen containing compounds** | |  |  |  |  |  |  |
|  | 2-Methylbutanenitrile | 0.773 | 0.862 | 0.649 | 0.89 | 1 | 0.994 |
|  | Methylpyrazine | 0.649 | 0.547 | 0.818 | 0.89 | 1 | 0.994 |
|  | Benzyl cyanide | 1 | 0.862 | 0.937) | 0.89 | 1 | 0.994 |
| **Fatty acid derivatives** | |  |  |  |  |  |  |
|  | (*Z*)-3-Hexen-1-ol | 0.832 | 1 | 0.649 | 0.89 | 1 | 0.994 |
|  | (*Z*)-3-Hexen-1-ol, acetate | 0.724 | 0.662 | 0.672 | 0.89 | 1 | 0.994 |
|  | (*Z*)-3-Hexen-1-ol, isobutyrate | 1 | 0.662 | 0.991 | 0.89 | 1 | 0.994 |
|  | (*Z*)-3-Hexen-1-ol, 2-methylbutanoate | 1 | 0.67 | 0.649 | 0.89 | 1 | 0.994 |
|  | (*Z*)-3-Hexen-1-ol, 3-methylbutanoate | 1 | 0.856 | 0.86 | 0.89 | 1 | 0.994 |
|  | (*Z*)-3-Hexen-1-ol tiglate | 1 | 0.76 | 0.649 | 0.89 | 1 | 0.994 |
|  | (*Z*)-3-Hexen-1-ol, benzoate | 0.87 | 0.694 | 0.649 | 0.89 | 1 | 0.994 |
| **Benzenoids or/and phenylpropanoids** | |  |  |  |  |  |  |
|  | Anisole | 1 | 0.862 | 0.649 | 0.89 | 1 | 0.994 |
|  | p-Methylacetophenone | 0.832 | 0.856 | 0.89 | 0.89 | 1 | 0.994 |
|  | o-Hydroxyacetophenone | 1 | 0.967 | 0.937 | 0.89 | 1 | 0.994 |
|  | 2-Phenethyl formate | 0.936 | 0.723 | 0.731 | 0.89 | 1 | 0.994 |
|  | 2,3-dimethylbenzofuran | 1 | 0.746 | 0.758 | 0.89 | 1 | 0.994 |
|  | 3-Ethylacetophenone | 0.649 | 0.662 | 0.937 | 0.89 | 1 | 0.994 |
|  | Methyl salicylate | 1 | 0.662 | 0.917 | 0.994 | 1 | 0.994 |
|  | Cuminal | 0.865 | 0.856 | 0.649 | 0.962 | 1 | 0.994 |
|  | 2-Phenylethyl acetate | 1 | 0.647 | 0.89 | 0.89 | 1 | 0.994 |
|  | (*E*)-3-Phenylpropenal | 0.936 | 0.746 | 0.781 | 0.89 | 1 | 0.994 |
|  | 3-Phenyl-3-pentanol | 0.994 | 0.746 | 0.818 | 0.89 | 1 | 0.994 |
|  | 2,3,6-Trimethylbenzaldehyde | 1 | 0.982 | 0.72 | 0.89 | 1 | 0.994 |
|  | Olivetol, dimethyl ether | 1 | 0.746 | 0.888 | 0.89 | 1 | 0.994 |

^a^ *P* value of Student’s *t*-test or Wilcoxon rank-sum test (for compounds that meet assumptions of normality or compounds that do not meet these assumptions, respectively).

^b^ DMNT = 4,8-Dimethylnona-1,3,7-triene.

^c^ TMTT = 4,8,12-Trimethyl-1,3,7,11-tridecatetraene.
